# Cohort Study: Central Venous Catheter-Related Complications in Children with Hematologic Diseases at a Single Center

**DOI:** 10.4274/tjh.2013.0403

**Published:** 2015-05-08

**Authors:** Ayhan Pektaş, Ateş Kara, Aytemiz Gurgey

**Affiliations:** 1 Afyon Kocatepe University Faculty of Medicine Hospital, Department of Pediatrics, Afyonkarahisar, Turkey; 2 Hacettepe University Faculty of Medicine, İhsan Doğramacı Children’s Hospital, Clinic of Pediatric Infectious Diseases, Ankara, Turkey; 3 Hacettepe University Faculty of Medicine, İhsan Doğramacı Children’s Hospital, Clinic of Pediatric Hematology, Ankara, Turkey

**Keywords:** Blood Coagulation, Hematologic manifestation, Infection, Pediatric leukemia

## Abstract

**Objective:**

This study aims to document and analyze the central venous catheter (CVC)-related complications in children with hematological diseases who were treated within a single institution.

**Materials and Methods:**

A retrospective investigation was conducted in 106 pediatric patients in whom 203 CVCs were inserted. A total of 175 catheter-related complications occurred in 5 years.

**Results:**

The rates of clinical catheter infections, local catheter infections, venous thromboembolism, bleeding, and mechanical complications were 2.6, 1.1, 0.2, 0.2, and 0.2 per 1000 catheter days. Methicillin-resistant Staphylococcus epidermidis was the predominant infectious organism in blood and catheter cultures. The children with leukemia had a significantly higher frequency of clinical catheter infections (p=0.046). The children who underwent bone marrow transplantation had a significantly lower frequency of clinical catheter infections (p=0.043) and higher frequency of local catheter infections (p=0.003). The children with implanted catheters had a significantly lower frequency of clinical catheter infections (p=0.048). The children with thrombocytopenia had significantly fewer local catheter infections and significantly more clinical catheter infections and catheter-related bleeding (respectively p=0.001, p=0.042, and p=0.024).

**Conclusion:**

Leukemia, bone marrow transplantation, and thrombocytopenia are risk factors for CVC-associated complications. The relatively higher number of interventions performed via permanent catheters may be responsible for the significantly increased incidence of systemic infections and mechanical injury.

## Abstract (tr)

**Amaç:**

Bu çalışma, tek bir merkezde hematolojik hastalık nedeniyle tedavi edilen çocuklarda santral venöz kateter (SVK) kullanımıyla ilişkili olarak ortaya çıkan komplikasyonları belirlemeyi ve incelemeyi amaçlamaktadır.

**Gereç ve Yöntemler:**

Beş yıl boyunca, 203 SVK uygulaması yapılan 106 çocuk hastada ortaya çıkan 175 katetere bağlı komplikasyon geriye dönük olarak değerlendirilmiştir.

**Bulgular:**

Klinik kateter enfeksiyonu, lokal kateter enfeksiyonu, venöz tromboembolizm, kanama ve mekanik komplikasyon oranları, sırasıyla, 1000 kateter gününde 2,6, 1,1, 0,2, 0,2 ve 0,2 olarak hesaplanmıştır. Metisiline dirençli Staphylococcus epidermidis, kan ve kateter kültürlerinden en çok izole edilen mikroorganizmadır. Lösemili çocuklarda klinik kateter enfeksiyonlarının sıklığı anlamlı olarak yüksektir (p=0,046). Kemik iliği transplantasyonu yapılan çocuklarda klinik kateter enfeksiyonu sıklığı anlamlı olarak düşük (p=0,043) bulunurken lokal kateter enfeksiyonu sıklığı anlamlı olarak yüksektir (p=0,003). İmplante kateteri olan çocuklarda klinik kateter enfeksiyonlarının sıklığı anlamlı olarak düşüktür (p=0,048). Trombositopenisi olan çocuklarda lokal kateter enfeksiyonları anlamlı olarak daha az görülürken klinik kateter enfeksiyonları ve katetere bağlı kanama anlamlı olarak daha sık ortaya çıkmıştır (sırasıyla p=0,001, p=0,042 ve p=0,024).

**Sonuç:**

Lösemi, kemik iliği transplantasyonu ve trombositopeni, SVK ile ilişkili olarak çocuklarda ortaya çıkan komplikasyonlar için en önemli risk etkenleridir. Kalıcı kateterler aracılığıyla gerçekleştirilen müdahale sayısındaki artış, sistemik enfeksiyon ve mekanik hasar riskindeki anlamlı yükselişten sorumlu olabilir.

## INTRODUCTION

Central venous catheters (CVCs) are clinical tools of the utmost importance that facilitate the administration of chemotherapy, antibiotics, blood products, fluids, and parenteral nutrition and the collection of blood samples in children with hematological diseases [1]. 

Tunneled, cuffed, silastic CVCs were first introduced by Broviac and colleagues and were improved subsequently by Hickman and colleagues. Afterwards, totally implantable vascular access devices (ports) were developed. These devices require less frequent care and provide more freedom and comfort for the patients [2,3].

Despite all preventive measures, systemic and local infections remain a challenge for the utilization of CVCs, including ports and external lines with the expanding use of ports [4]. Venous thromboembolism (VTE) is another serious catheter-related complication that usually appears in critically ill children [5,6]. Access-related bleeding and mechanical complications such as blockage, leakage, dislodgement, and malposition are also encountered in children with catheters [7]. 

The present study aims to document and analyze the CVC-related complications in pediatric hematology patients who were treated within a single institution over a period of 5 years. Since CVCs have indispensable importance in clinical practice, possible precautions for the prevention of relevant complications are also discussed.

## MATERIALS AND METHODS

### Selection of Patients

The inclusion criteria were the diagnosis of hematological diseases and the administration of CVCs in pediatric patients aged less than 18 years at the study center between June 2003 and December 2007. Therefore, 136 children were initially chosen for the study. Three children with a documented infection or VTE in any location within 6 weeks, 3 children for whom CVCs were implanted at the site of a previously confirmed infection or VTE, 2 children with concurrent anticoagulant treatment, 2 children with sensitivity to antibiotics and/or anticoagulants, 3 children with bacterial endocarditis, 2 children with severe thrombocytopenia (<20x109/L), and 5 children with severe hypertension, renal dysfunction, or hepatic disease were excluded [8]. Thus, 106 children for whom 203 CVCs were inserted were finally enrolled into the study. A total of 175 catheter-related complications were analyzed retrospectively. Ethical committee approved this study.

### Devices and Their Use

All CVC implantations were performed under general anesthesia and sterile conditions by experienced pediatric surgeons or interventional radiologists. The CVCs were inserted via the internal jugular vein using an infraclavicular approach, namely the Seldinger technique. No perioperative antimicrobial prophylaxis was administered and no serious perioperative complications were observed except one case of hemothorax. The position of the catheter tip was verified with an on-table chest X-ray after the insertion was completed. Either titanium-based polysulfone ports with a single lumen or multilumen tunneled catheters were used. When port failure occurred, multilumen tunneled catheters were implanted until a new port could be relocated. The catheter exit site dressings were changed each Monday, Wednesday, and Friday of the week or when the dressing became contaminated or wet. All dressing changes were done with an aseptic technique using a mask and sterile gloves. The site was cleaned with 10% povidone-iodine and covered with a sterile dressing. The catheter was flushed every other day with a standardized procedure using 3 mL of heparin/normal saline solution (100 U/mL), which was prepared daily. The catheter cap was prepared by 10% povidone-iodine application before each needle entry and was changed weekly or as needed. The ports were accessed at least once every 4 weeks using sterile techniques and during periods of prolonged, continuous use, while the needles were changed every 7 days and tubing was renewed every 3 days. Catheter care was performed by trained doctors.

### Definitions

Clinical catheter infection is defined as fever of ≥38.5 °C without any obvious cause of fever and rigors associated with flushing of catheters without microbiologic documentation. Proven catheter infection refers to 2 catheter cultures positive for coagulase-negative Staphylococcus but negative peripheral blood culture or a positive culture for any other microorganism. Catheter-associated bacteremia denotes positive peripheral and central blood cultures. Bacteremia unrelated to catheter means a negative catheter culture but a positive peripheral culture. Catheter-related local infection refers to inflammation (redness, edema, warmth, tenderness, discharge) around the site or along the catheter tunnel [9,10]. 

Mechanical complications are any malposition or extravasation, while hematological complications include bleeding, hematoma, or thrombosis at the site of insertion. The diagnosis of VTE was based on the detection of thrombi and/or flow absence in noncompressible veins by Doppler ultrasonography [11].

Absolute indications for CVC removal were mechanical complications, successful completion of chemotherapy, tunnel or pocket infections, persistence of fever and positive blood cultures obtained later than 48 h after the initiation of appropriate antimicrobial therapy, septic emboli, and persistent obstruction or thrombosis of a large vein refractory to thrombolysis. 

### Data Collection

The medical records of the eligible patients were reviewed by the first author to obtain data on age, sex, primary diagnosis, duration of catheterization, indication of catheterization, catheter type, catheter-related complications, and clinical outcomes of these complications. There were no emergency catheter insertions.

### Statistical Analysis

Collected data were analyzed with SPSS 11.5 (SPSS Inc., Chicago, IL, USA). Data distribution was tested by the Smirnov-Kolmogorov test. Data were expressed as mean±standard deviation or percent where appropriate. Parametric variables of 2 groups were compared by independent-samples t-test, whereas those of 3 groups were compared by the one-way ANOVA. Nonparametric variables of 2 groups were analyzed by Mann-Whitney U test and those of 3 groups were evaluated by the Kruskal-Wallis test and Pearson chi-square test. If the one-way ANOVA or other tests resulted in statistical significance, a post-hoc test was applied. P<0.05 was accepted to be statistically significant.

## RESULTS

The reviewed children had a mean age of 6.4 years (minimum-maximum: 0.2-17 years). Seventy-three children (68.9%) were boys and 33 children (31.3%) were girls. Acute lymphoblastic leukemia (50.9%, n=54) and acute myeloid leukemia (14.2%, n=15) were the most common hematological diseases, followed by aplastic anemia (6.6%, n=7), myelodysplastic syndrome (4.7%, n=5), hemophagocytic syndrome (4.7%, n=5), Fanconi aplastic anemia (5.7%, n=6), hemolytic uremic syndrome (4.7%, n=5), thalassemia major (3.8%, n=4), chronic myeloid leukemia (2.8%, n=3), and juvenile myelomonocytic leukemia (1.9%, n=2).

The CVCs were either tunneled (55.2%, n=112) or implanted (44.8%, n=91). These vascular access devices were applied to administer chemotherapy (59.1%, n=120), bone marrow transplantation (27.1%, n=55), drug infusion (6.9%, n=14), total parenteral nutrition (5.4%, n=11), and plasmapheresis (1.5%, n=3). 

The total and mean durations of catheterization were 40,162 and 378.9 days (minimum-maximum: 1-1460 days), respectively. The rates of clinical catheter infections, local catheter infections, VTE, bleeding, and mechanical complications were 2.6, 1.1, 0.2, 0.2, and 0.2 per 1000 catheter days.

Methicillin-resistant Staphylococcus epidermidis was the predominant infectious organism, isolated in 48 of 150 catheter cultures (32.0%) and 19 of 150 blood cultures (12.7%). Catheter cultures also yielded Candida albicans (6.7%, n=10), Staphylococcus saprophyticus (6.7%, n=10), and methicillin-resistant Staphylococcus aureus (6.0%, n=9). Moreover, Escherichia coli (4.7%, n=7), methicillin-sensitive Staphylococcus aureus (2.7%, n=4), and Staphylococcus epidermidis (2.7%, n=4) were detected in blood cultures. About 29.1% of the CVCs (59 of 203) were removed due to catheter-related complications. The most frequent causes for catheter removal were infections (42.4%) and mechanical injury (57.6%). No deaths occurred due to catheter-related complications.

When compared to 37 patients with other hematological diseases, 69 patients with leukemia had significantly longer duration of catheterization (p=0.001) and higher frequency of clinical catheter infections (p=0.046) (Table 1). When compared to the remaining patients, 28 children who underwent bone marrow transplantation had significantly shorter duration of catheterization (p=0.001), lower frequency of clinical catheter infections (p=0.043), and higher frequency of local catheter infections (p=0.003) (Table 2). When compared to 58 children with tunneled catheters, 48 children with implanted catheters had significantly longer duration of catheterization (p=0.001) and lower frequency of clinical catheter infections (p=0.048) (Table 3). When compared to 34 patients with normal platelet counts, 72 patients with thrombocytopenia had significantly lower local catheter infections (p=0.001) and significantly higher frequency of clinical catheter infections and catheter-related bleeding (p=0.042 and p=0.024) (Table 4).

## DISCUSSION

Despite the widespread utilization of broad-spectrum antibiotics, infections remain a significant cause of morbidity related to catheters. European studies documented that the majority of patients with bloodstream infections had catheters and defined catheter insertion as an independent risk factor for sepsis [12]. A study by Ertem et al. reported that the rate of catheter-related sepsis was 4.9 per 1000 catheter days in children with right atrial catheters [13]. In a similar study, the rate of catheter-related infection was 2.5 per 1000 catheter days in children with totally implantable CVCs [14]. A recent study indicated the rate of catheter-associated bloodstream infections as 7.4 per 1000 catheter days in children [15].

The present study indicates clinical and local infections as the most frequently encountered catheter-related complications. The rates of clinical catheter infection and local catheter infection were respectively 2.6 and 1.1 per 1000 catheter days, corresponding to an overall rate of 3.7 episodes per 1000 catheter days. Clinical catheter infections are found to be associated with leukemia, utilization of tunneled catheters, and thrombocytopenia, whereas local catheter infections are related to bone marrow transplantation. The relatively lower rate of catheter-related infections in this study may be attributed to the adoption of highly sterile minimal-touch techniques for catheter insertion, care, and removal. 

Coagulase-negative staphylococci are responsible for the majority of catheter-related bloodstream infections, whereas gram-negative bacteria, enterococci, and Candida species account for the remainder of these infections [12,16]. Ertem et al. also designated coagulase-negative staphylococci and Candida species as the most common organisms, respectively accounting for 25.0% and 13.1% of catheter-related infections in children with right atrial catheters [13]. Another study reported that gram-positive bacteria (predominantly coagulase-negative staphylococci) were present in 55% of all infections associated with totally implantable CVCs, but no fungi were detected in blood or catheter cultures [14]. Celebi et al. documented that coagulase-negative Staphylococcus was isolated in 41% of catheter-related bloodstream infections [15]. In accordance with the literature, Staphylococcus epidermidis was the predominant infectious organism in blood and catheter cultures reviewed in this study. However, Staphylococcus epidermidis was specified in only 32% of blood cultures and 13% of catheter cultures. The relatively lower incidence of staphylococcal infection may be caused by the differences in the hematological diagnoses and catheter types. 

Catheter-related VTE is a serious complication that usually affects critically ill children in whom catheters are applied via the left subclavian vein and used for a prolonged period of time [17,18,19,20,21]. The rate of catheter-related VTE was 0.2 episodes per 1000 days in this study. Meticulous catheter care combined with the use of thrombolytic agents and relatively shorter duration of catheterization may be the underlying reasons for this relatively lower rate. Due to the low rate of catheter-related VTE, no risk factors could be identified for this complication.

Previously published studies have addressed thrombocytopenia as a risk factor for catheter-related bleeding. This complication can be prevented by visualizing the catheter route and applying tamponade at the insertion site [19,22]. The rate of catheter-associated bleeding was 0.2 episodes per 1000 days in this study. Although this is a relatively low number, thrombocytopenia also emerges as a risk factor for catheter-related bleeding. 

A thorough review of the literature demonstrates that up to 67% of inserted catheters are removed for various reasons. When deaths and elective removals are excluded, the most frequent causes of catheter removal are infection and mechanical complications, including dislodgement, leakage, occlusion, and malposition [3,11]. Approximately 88% of right atrial catheters were removed and the most common reasons for removal were infection (42.4%) and dislodgement (32.2%) in a study by Ertem et al. [13]. Another study reported that 29% of all totally implantable catheters were removed due to infections and mechanical complications [14]. Celebi et al. showed that nearly 20% of catheters were removed and the most frequent reasons for removal were infections (44.4%) and mechanical complications (55.6%) [15]. As for the present study, 29% of the CVCs were removed due to catheter-related complications. The most frequent causes for catheter removal were infections (42.4%) and mechanical injury (57.6%). These figures are in accordance with the literature.

Leukemia has been identified as a major risk factor for catheter-associated infections [17,23]. Although several studies were unable to detect such a relationship [14,22], this study also describes leukemia as an underlying factor for catheter-related infections. Leukemia precipitates qualitative and quantitative blood cell abnormalities. Furthermore, chemotherapy administered for leukemia can cause immunosuppression and thus induce infections.

Bone marrow transplantation was determined as an independent risk factor for catheter-related bloodstream infections [24]. In contrast, this study shows that bloodstream infections were significantly less frequent and local infections were significantly more frequent in children undergoing bone marrow transplantation. Such a discrepancy may be caused by the utilization of implanted catheters in children undergoing bone marrow transplantation.

Implanted catheters have been designed to lessen the need for special care and to facilitate body movements. The children with implanted catheters were less likely to develop infections and VTE, whereas mechanical complications increased in children who had implanted catheters for the treatment of hematological diseases [24,25]. 

Table 5 summarizes the findings of previous clinical studies that evaluated the infectious complications in children with implanted catheters [22,23,24]. Variations within study samples and designs are the major confounding factors for the interpretation of the results yielded by the present study and previously published studies. The adoption of different criteria and definitions by different health centers might have contributed to this discrepancy, as well. This study evaluates all catheter-related complications globally and aims to help pediatricians perceive the potential hazards of CVC application in children as a whole. However, its power is limited by its retrospective design and relatively small cohort size. 

Leukemia, bone marrow transplantation, and thrombocytopenia are risk factors for CVC-associated complications. In order to avoid catheter-related complications, previously established guidelines for catheter insertion, care, and removal should be followed carefully. Further research is warranted to clarify the risk factors for catheter-related complications. 

## Figures and Tables

**Table 1:**
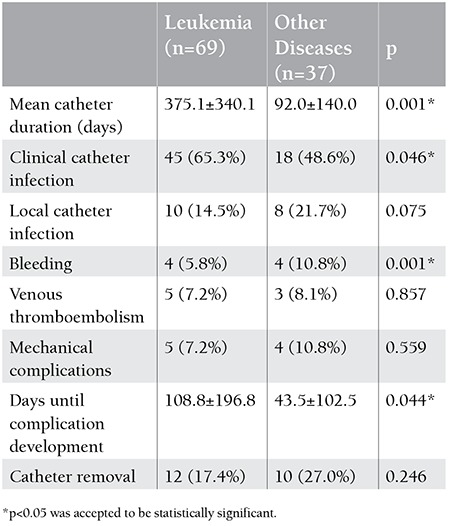
Catheter complications in patients with leukemia and patients with other hematological diseases.

**Table 2:**
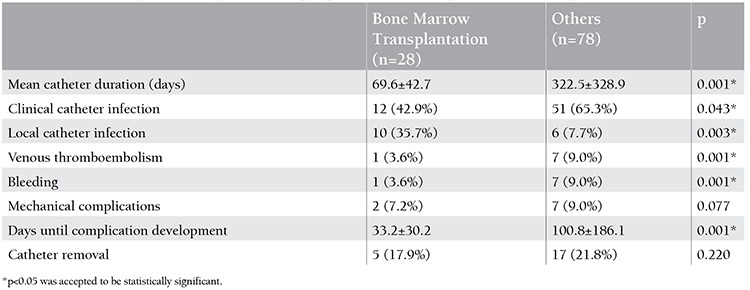
Catheter complications in patients undergoing bone marrow transplantation and other patients.

**Table 3:**
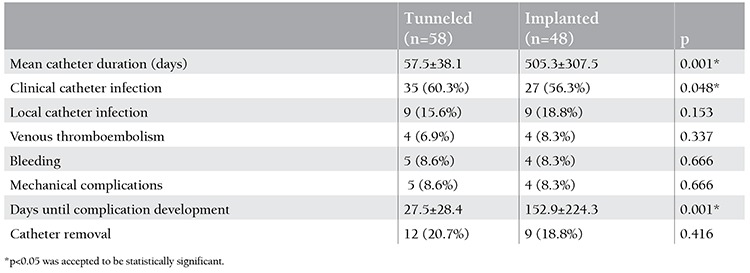
Catheter complications in patients with tunneled and implanted catheters.

**Table 4:**
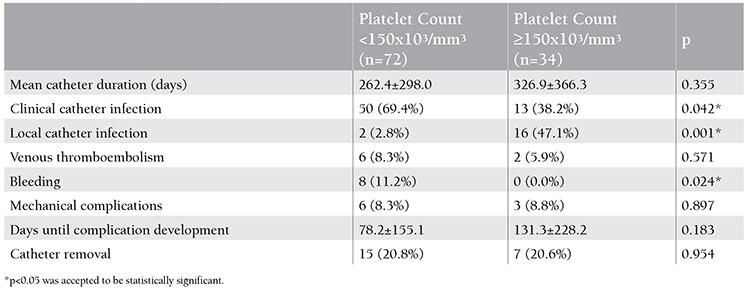
Catheter complications in patients with low and normal platelet counts.

**Table 5:**
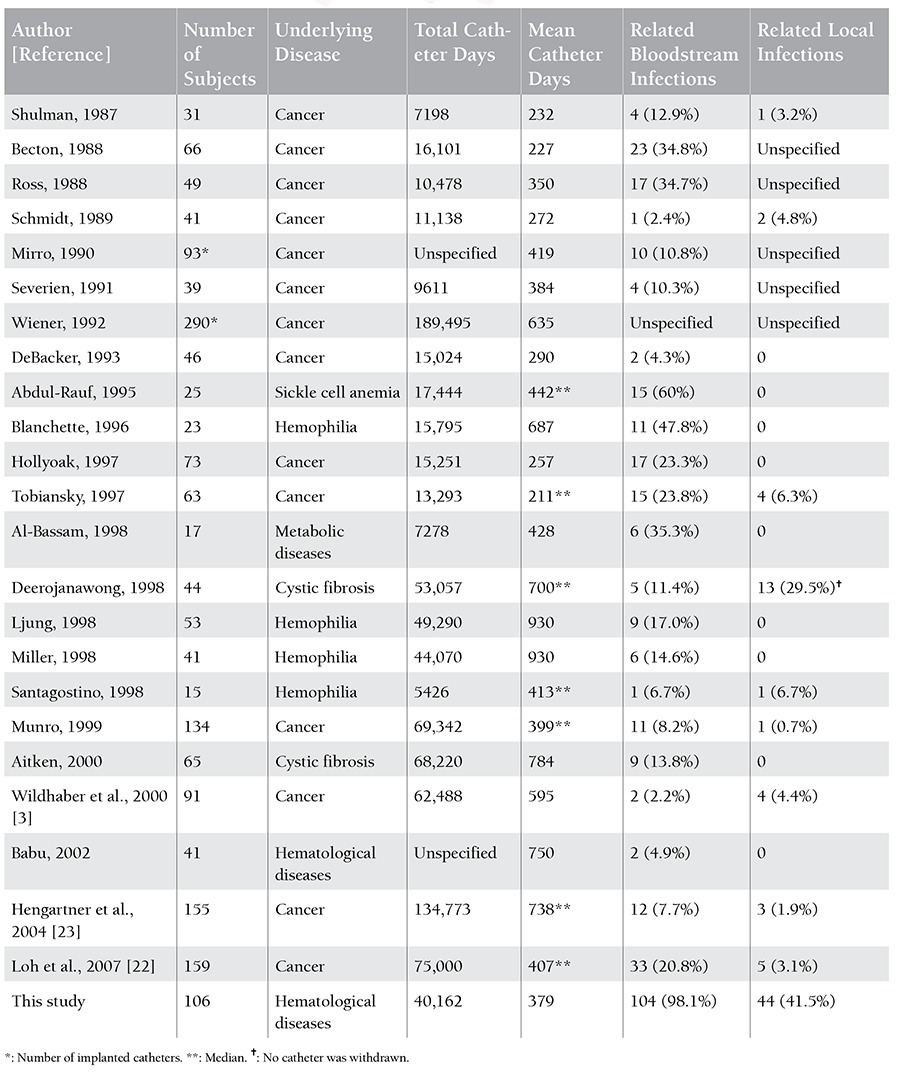
Literature review of infectious complications of implanted port catheters in children.
